# Molecular screening for avirulence alleles *AvrLm1* and *AvrLm6* in airborne inoculum of *Leptosphaeria maculans* and winter oilseed rape (*Brassica napus*) plants from Poland and the UK

**DOI:** 10.1007/s13353-014-0235-8

**Published:** 2014-08-01

**Authors:** Joanna Kaczmarek, Akinwunmi O. Latunde-Dada, Witold Irzykowski, Hans J. Cools, Jenna F. Stonard, Andrzej Brachaczek, Malgorzata Jedryczka

**Affiliations:** 1 Institute of Plant Genetics, Polish Academy of Sciences, Poznan, Poland; 2 Rothamsted Research, Harpenden, Hertfordshire AL5 2JQ UK; 3 Syngenta’s International Research Centre, Jealott’s Hill, Bracknell, Berkshire RG42 6EY UK; 4 DuPont Poland, Postepu 17b, 02-676 Warszawa, Poland

**Keywords:** Airborne inoculum, Air sampling, Avirulence genes, Disease forecasting, *Leptosphaeria maculans*, Phoma stem canker

## Abstract

A combination of staining, light microscopy and SYBR green- and dual-labelled fluorescent probe-based qPCR chemistries with species- and gene-specific primers was employed to evaluate fluctuations in the aerial biomass of *Leptosphaeria maculans* spores captured by volumetric spore trappings in Poznan, Poland (2006, 2008) and Harpenden, UK (2002, 2006). Arising from these surveys, DNA samples extracted from Burkard spore-trap tapes were screened for fluctuation patterns in the frequencies of *AvrLm1* and *AvrLm6*, the most prominent of the 15 genes that code for avirulence effectors in this Dothideomycete cause of the destructive phoma stem canker disease of oilseed rape worldwide. In Poznan, very low frequencies of *AvrLm1* allele were found in the autumn of both 2006 and 2008, reflecting significantly increased cultivation of rape seed with *Rlm1*-based resistance. In contrast, at least six folds-higher frequencies of *AvrLm6*, which were also confirmed by end-point PCR bioassays on phoma-infected leaves from the same region of Poland, were obtained during both years. In the UK, however, relatively higher *AvrLm1* allele titres were found in *L. maculans* spores captured in air samples from the autumn of 2002 on the experimental fields of Rothamsted Research, Harpenden, that were historically sown to genetically heterogeneous *B. napus* cultivars. In the 2006 screen these levels had plummeted, to a 1:4 ratio, in favour of frequencies of the *AvrLm6* allele. Patterns of fluctuations in *erg11* (*CYP51*) fragments coding for sterol 14α-demethylase suggest October as the month with the most viable wind-dispersed *L. maculans* propagules of each season of the screens.

## Introduction

Various techniques for spore capture are increasingly being used in supporting decisions on the protection of agricultural crops against important pathogens causing great yield losses. The systems mostly used are volumetric traps that enable a continuous monitoring of fungal spore concentrations in known volumes of air samples following capture. Advice based on spore counts is done routinely in several countries, including USA (Isard et al. 2007), Australia (Blackleg Sporacle, Salam et al. 2003), India (Devi and Singh 2007) and the UK (for the development of PASSWORD; Gladders et al. 2006). Currently in Europe, the biggest monitoring system based on spore counts is the System for Forecasting Disease Epidemics (SPEC; Jedryczka et  al. 2004, 2012) which was designed in Poland for monitoring the aerial dynamics of ascospore concentrations of the pathogen *Leptosphaeria maculans*, the cause of the phoma stem canker disease of oilseed rape. This damaging disease is prevalent on brassicas worldwide (Fitt et  al. 2006), except for China and India, where agricultural practices and weather conditions appear to prevent epidemics of this destructive pathogen.

The species *L. maculans*, usually found in association with the less damaging *L. biglobosa* (cause of phoma leaf and upper stem necrosis), has become in recent years one of the most widely-studied fungal species (Rouxel and Balesdent 2005; Kaczmarek and Jedryczka 2011). Apart from causing economic yield losses, key features of the biology of this ascomycete fungus make it a candidate for genetic and molecular biological studies. Pseudothecia, the fruiting bodies, of *L. maculans* are easily formed in natural conditions as well as in controlled environment thereby permitting studies on the prolonged haploid phase of the life cycle and easy separation (by pulsed gel electrophoresis) of its 17 chromosomes (Cozijnsen et al. 2000), including a dispensable minichromosome (Leclair et al. 1996; Balesdent et al. 2013). The pathogen is easily cultured on artificial media and produces numerous conidial spores with identical genetic material. Recent successful transformations of *L. maculans* with green fluorescent protein (GFP), as well as with DS-Red, reporter gene constructs have made it possible to study the endophytic growth of the pathogen within asymptomatic host tissues (Sexton and Howlett 2001; Eckert et al. 2005). More recently, the genome of this dothideomycete fungus was sequenced and the annotations of fungal avirulence genes have been released publicly (Rouxel et al. 2011).

Knowledge of the distribution of avirulence genes within fungal populations is crucial for choosing the strategies of crop protection against pathogens. As interactions between products of avirulence genes and products of host plant resistance genes determine disease outcome, studies designed to monitor avirulence genes are of great importance in planning both general and specific strategies of disease managements, including the choice of cultivars that are most suitable for resisting pathogen populations as well as planning breeding strategies for future control of the disease. In total, 11 avirulence genes comprising *AvrLm1*-*AvrLm11* (including *AvrLm4*-*7*) in *L. maculans* and corresponding to 11 *R* genes viz, *Rlm1*-*Rlm11* in brassica (*B. napus*, *B. juncea*, *B. nigra* and *B . rapa*) host plants (Ansan-Melayah et al. 1995, 1998; Balesdent et al. 2001, 2002, 2005, 2006, 2013; Delourme et al. 2006; Kutcher et al. 2010; Soyer et al. 2014) have been identified so far. Four other resistance genes, *LepR1*, *LepR2*, *LepR3* and *LepR4* were identified in Canadian canola species (Rimmer 2006; Larkan et al. 2013; Yu et al. 2013).

*Leptosphaeria maculans* has a high evolutionary potential, according to the criteria of McDonald and Linde (2002), arising from a combination of its possession of both sexual and asexual cycles, an effective wind-dispersal mechanism for ascospores and rain-splash method for the infective conidia, as well as its large population size. In the recent past, rapid degradation and loss (‘breakdown’) of qualitative resistance have attended the large scale cropping of oilseed rape cultivars possessing a single vertically-resistant *Rlm* gene (Gout et al. 2006). For example, the cultivar ‘Surpass 400’ (specific resistance derived from *B. rapa* spp. *sylvestris*; Crouch et al. 1994) which was introduced to Australia in 2000 and was widely adopted by rapeseed farmers, became no longer effective by 2003 against *L. maculans* populations, causing severe losses to the oilseed rape industry (Li and Cowling 2003) thereby. Similarly, the large scale cropping from 1996 to 1999 (43.7 % of total area grown), of oilseed rape with the *Rlm1* gene in France, resulted in a pathogen population change (increase in the frequency of *avrLm1*) that rendered ineffective, by the 2000/2001 growing season, this qualitative resistance/trait (Rouxel et al. 2003). However, Van de Wouw et al. (2009) have suggested that recognition of avirulence in *L. maculans* towards a *Brassica napus* cultivar with ‘*sylvestris*-derived’ resistance involved two resistance genes. Resistance gene *Rlm6* currently appears to provide effective resistance to *L. maculans*. Stachowiak et al. (2006) identified *AvrLm6* in 100 % of European isolates examined in 2002, whereas *AvrLm1* ranged between 1.2 % in Teendorf (Germany) to 17.6 % in Harpenden (UK, England). The most recent study on *AvrLm1* and *AvrLm6* in the UK was done in 2006–2009 and found that while *AvrLm1* allele frequencies remained low those of *AvrLm6* had decreased to 35–66 % (Van de Wouw et al. 2010). In a large-scale survey of French *L. maculans* in 2001 and 2002 all isolates of *L. maculans* possessed *AvrLm6* (Balesdent et al. 2006). A similar pattern was reported for North America, where *AvrLm6* was present in the majority (75–100 %) of isolates, with the exception of a field in Camrose, Alberta (Canada), where it was found to be below 40 % (Dilmaghani et al. 2009). The presence of avirulence gene *AvrLm1* in North America appears to depend on both location and popularity and expanse of oilseed rape cultivation; in Mexico the allele was detected in all isolates of *L. maculans* sampled, whereas in the majority of fields in Alberta and Saskatchewan, Canada, *AvrLm1* was completely absent. Strangely, however, this allele was also very low (1.5 %) in Chile, where oilseed rape is not popularly cultivated.

In recent reports (Kaczmarek et al. 2009, 2012; Jedryczka et al. 2010b; Karolewski et al. 2012; Atkins et al. 2013) we have presented studies that demonstrated by quantitative PCR techniques the detection, discrimination and quantification of seasonally captured propagules of *Leptosphaeria* spp, *Pyrenopeziza brassicae* and *Sclerotinia sclerotiorum* in air samples from oilseed rape fields of Poland and southern UK. These studies utilized Burkard 7-day air samplers, primers based on internal transcribed spacer (ITS) region or *β*-tubulin gene fragment sequences and SYBR-green or dual-labelled fluorescent probe chemistry to monitor seasonal fungal abundance in air spora ahead of the onset of damaging symptoms of diseases caused by these pathogens. In the current study, we seek to further demonstrate the possibilities of monitoring, screening and characterizing these spores for viability and the presence of virulence and pathogenicity effector genes. A report (Van de Wouw et al. 2010) has demonstrated the determination of frequencies of avirulence alleles in airborne inoculum of *L. maculans*.

The aim of this study was to screen for the proportions of *AvrLm1* and *Avrlm6* avirulence genes in *L. maculans* propagule populations captured in autumnal air samples collected during the 2006/2007 and 2008/2009 seasons from Great Poland, one of the main oilseed rape growing areas of Poland. The relative abundance of *AvrLm1* and *Avrlm6* avirulence alleles in *L. maculans* aerial propagule populations sampled in Hertfordshire, UK during the autumns of the 2002/2003 and 2006/2007 oilseed rape seasons was also determined for comparison. These studies were undertaken primarily to assess the minimum detection limits for concentrations of fungal DNA in a sample that would enable the detection of avirulence gene fragments and whether this resolution might be put to practical use. The proportions of avirulence alleles in spores were compared to the ratio of these alleles in leaf explants that were sampled from field-sown plants after natural phoma lesion infection. In addition, *L. maculans* spore populations were evaluated by light microscopic counts and abundance determination was assessed by real-time, quantitative PCR employing either SYBR green or dual-labelled fluorescent dye chemistry. Azoles, the largest group of sterol 14α-demethylation inhibiting fungicides (Cools et al. 2013), are extensively used in European rapeseed cultivation, particularly in Poland (Jedryczka et al. 2012) and the UK (Fitt et al. 2006), for the field control of phoma stem canker and foliar diseases (Carter et al. 2013). The sterol enzyme (14α-demethylase; ERG11, a cytochrome P450) inhibited by these durable fungicides (imidazoles and triazoles) is pivotal to ergosterol biosynthesis, essential for fungal plasma membrane function and a determinant of fungal growth and viability (Weete et al. 2010). To further evaluate fungal biomass and ascertain the potential for fungal viability and active growth, spores were also screened for the presence of fragments of *Lmac erg11* (Griffiths and Howlett 2002) the sterol 14α-demethylase (=CYP51) gene from *L. maculans*.

## Materials and methods

### Ascospore sampling

Sampling of the spores of *Leptosphaeria* spp. was done in the autumns of 2006 and 2008 in Poznan, Poland using a Hirst type seven-day volumetric spore sampler (Burkard Manufacturing Co., Rickmansworth, UK). The trap was located at the grounds of the Institute of Plant Genetics, the Polish Academy of Sciences in Poznan in Poland. The spore trap was surrounded by oilseed rape stubble infected with stem canker which had been collected from the local fields after harvest in July 2006 and 2008. The spore sampler was operated according to the instructions of Lacey and West (2006). Weekly strips of Melinex tape were divided into daily pieces (14 × 48 mm). These were then cut longitudinally, with one piece used for extraction of DNA whilst the other was mounted onto a microscope slide, stained with 0.1 % (*w*/*v*) trypan blue in lactophenol and examined with a light microscope under 400× magnification (Zeiss Axiostar, Germany). The numbers of spores present on tapes were re-calculated to daily ascospore numbers per 1 m^3^ of air. A similar sampling and detection protocol was done in the autumn of the 2002/2003 and of the 2006/2007 oilseed rape seasons at Rothamsted Research in Harpenden, Hertfordshire, UK.

### Sampling and isolation of pathogens from plant tissues

The isolates characterized in this study originated from field experiments established in autumn 2008 at four sites in Poland in Great Poland (Cerekwica, Gora, Nowa Wies Ujska, Pawlowice). The field plots were sown with the winter cultivar of oilseed rape: Bosman (Plant Breeding Strzelce) in Cerekwica and Winna Gora, PR46W10 (Pioneer Hi-Breed) in Nowa Wies Ujska and Pawlowice. In total one hundred leaves with visible symptoms of phoma leaf spotting were collected from rapeseed fields located in the region of study. Fragments of leaves with disease symptoms were surface-sterilized with 1 % sodium hypochlorite for 2 min followed by rinsing (3 times, 2 min) in sterile distilled water. Small sections of sterilized leaves were placed on potato dextrose agar (PDA, Sigma) medium supplemented with streptomycin sulphate (0.02 % *w*/*v*). Fungal isolates were subcultured until they were free of contaminants. A hyphal tip of each isolate was excised with a sterile needle and a binocular microscope and subcultured on to fresh PDA medium. The taxonomic identification was based on culture morphology and size of ITS products from end-point PCR amplification, as proposed by Williams and Fitt (1999).

Axenic cultures were kept at 22 °C for 12–14 days under alternating 12 h white/12 h near-UV light. Conidia of individual isolates of *L. maculans* were suspended in sterile double-distilled water; the suspensions were adjusted to a concentration of 10^7^ conidia ml^−1^ and stored at−20 °C for further analysis.

### Cotyledon test

Fifty one isolates obtained from naturally-infected plants of oilseed rape in the region of Great Poland were studied using the cotyledon test, according to the method described by Stachowiak et   al. (2006). Three differential cultivars/lines were used to perform the test: Columbus possessing *Rlm1* and *Rlm3* resistance genes, line 22-1-1 with *Rlm3* and Goéland with *Rlm9* resistance gene (Balesdent et al. 2005). Moreover, the experiment included cv. Darmor-MX, with the *Rlm6* resistance gene, but with unknown *Rlm9* locus (Stachowiak et al. 2006). The avirulence genes were characterized based on the reaction of differential cultivars/line to artificial inoculation using the tested isolates. No reaction to inoculation or the formation of small dark ring around the infection site was recorded as a resistant reaction, identifying the isolate as avirulent, whereas formation of light green or pale beige leaf spots with pycnidia was recorded as susceptible reaction, resulting from the virulence of the isolate (Balesdent et al. 2001).

The test was done using 12-day old seedlings, cultivated at controlled environment room with 12 h photoperiod, with 20 °C at the light phase and 16 °C in darkness. Prior to inoculation the plants were watered to maintain high humidity. Each half of the cotyledon was punctured with a needle and then inoculated with a 10 μL droplet of inoculum suspension of 1 × 10^7^ conidia ml^−1^. Such a droplet was placed on each half of the cotyledon, so that one plant contained four inoculation sites. Each isolate was screened on all four cultivars with eight to ten plants. After inoculation, trays with plants were covered with a plastic propagator lid and kept in darkness for 60 h, following which plants were kept at alternating 12 h photoperiod and the temperature conditions described above. The humidity in the chamber was increased to 70 % by using a humidifier HumiDisk (Carel Deutschland GmbH, Germany). Host response was scored 14 days after inoculation, using a 0–6 scale, as established (Balesdent et al. 2001) and described by Stachowiak et al. (2006).

### DNA extraction from Melinex tape and isolates

Daily, Melinex tape sections (48 mm) were cut into sections using sterile forceps and scissors and placed into a 2 ml microfuge tube. To each of these tubes was added one scoop (approx. 150 mg) of sterile acid-washed Ballotini beads (425–600 μm diameter). Fungi were grown in 50-ml Czapek-Dox broth (Sigma) with yeast extract (Oxoid) for 7–14 days at 25 °C. Fresh mycelium was freeze-dried and homogenized. DNA was extracted from the tapes and from individual isolates grown in culture media using a method described by Kaczmarek et al. (2009). The intial homogenate was incubated at 70 °C and partitioned against an equal volume of a 24:1 mixture of chloroform and isoamyl alcohol. DNA was precipitated by chilling incubation at −20 °C with ethanol and sodium acetate (3 M, pH 5). DNA pellets were washed with ice-cold 70 % ethanol, dried, dissolved in 100 μL 1 mM TE (Tris HCl pH 7.5, 1 mM EDTA) buffer and stored at −20 °C.

### Quantitative PCR to assess proportions of DNA of each Leptosphaeria maculans in the spore samples

For quantitative PCR, a standard 20-μL reaction contained 5 μL template DNA, 200 nM forward primer, 180 nM reverse primer (Mahuku et al. 1996; Liu et al. 2006), 10 μL SYBR Green JumpStart Taq ReadyMix (Sigma, UK), 0.08 μL ROX internal reference dye (Sigma, UK) and 3.78 μL nuclease-free, sterile water. For this study, duplicate 10-μL assays were routinely done. Thermal cycling parameters for detection were 95 ºC for 2 min followed by 38 cycles of 95 ºC for 15 s, 60 ºC for 30 s and 72 ºC for 45 s. To ascertain the specificity of the procedure, a dissociation (melting) curve was done after the final amplification cycle by heating samples at 95 °C for 15 s, cooling to 60 °C for 1 min and then heating to 95 °C for 15 s. Fluorescence was measured continuously and standard curves were developed from the C_t_ values of the amplification of standard genomic DNA concentrations (10 ng μL^−1^ to 1 pg μL^−1^) from *L. maculans* mycelial cultures. *Leptosphaeria maculans* DNA from these tapes was also assessed and quantified by real-time PCR utilizing dual-labelled probes and species-specific primers based on the nucleotides sequences of *β*-tubulin gene fragments from *L. maculans* (Kaczmarek et al. 2012). For this chemistry, a 20 μL reaction volume contained 5 μL template DNA, 10 μL qPCR supermix (Invitrogen, UK), 200 nM forward primer and 200 nM reverse primer and 100 nM of the dual-labelled fluorescent probe that was targeted at the *β*-tubulin gene fragment of *L. maculans*, 0.08 μL 50× ROX reference dye (Invitrogen, UK) and 2.5 μL autoclaved distilled water. Amplification and product detection were done at 50 °C for 2 min, 1 cycle at 95 °C for 2 min, followed by 45 cycles at 95 °C for 15 s and 60 °C for 45 s, while recording changes in fluorescence at 60 °C during each cycle and using nuclease-free water (Sigma, UK) as the no-template control. A standard curve was generated by plotting the C_t_ value for each sample of a standard series of genomic DNA concentrations (10 ng uL^−1^ to 100 fg uL^−1^) extracted from mycelial cultures of *L. maculans*. All samples were collected in duplicate.

### Quantitative PCR to screen for alleles of the AvrLm1, AvrLm6 and erg11 genes in captured spore samples

Real-time PCR quantitation of *AvrLm1*, *AvrLm6* (Gout et al. 2006; Fudal et al. 2007) and *erg11* (Griffiths and Howlett 2002) alleles in *L. maculans* spore samples was done by running uniplex diagnostic reactions based on the SYBR Green chemistry. Primers AvrLm1F (5′-ATTTCCAGACGTTCCGAGTG-3′), AvrLm1R (5′-ACGTTGTAATGAGCGGAACC-3′), AvrLm6F (5′-CGCACGAAGGCAACTATGTA-3′), AvrLm6R (5′-GCTTTTGGAGTTGGTCATGG-3′), LmacERG11F (5′-AAACCCGCACACATAATCGAAG-3′) and LmacERG11R (5′-TTCTCGTCGTCCTTTTCGTC-3′) were designed and used. Each standard 20-μL reaction contained 5 μL template DNA, 0.6 μL forward primer, 0.54 μL reverse primer, 10 μL SYBR Green JumpStart Taq ReadyMix (Sigma, UK), 0.08 μL ROX internal reference dye (Sigma, UK) and 3.78 μL nuclease-free, sterile water. For this study, duplicate 10-μL assays were routinely done. Thermal cycling parameters for detection were 95 ºC for 2 min followed by 38 cycles of 95 ºC for 15 s, 60 ºC for 30 s and 72 ºC for 45 s. To ascertain the specificity of the procedure, a dissociation (melting) curve was done after the final amplification cycle by heating samples at 95 °C for 15 s, cooling to 60 °C for 1 min and then heating to 95 °C for 15 s. Fluorescence was measured continuously and standard curves were developed from the C_t_ values of the amplification of standard genomic DNA concentrations (10 ng μL^−1^ to 1 pg μL^−1^) from *L. maculans* mycelial cultures. The primer pairs were tested (T_a_ = 60 °C) for cross reactivity and specificity by end-point PCR against genomic DNA (10–100 ng uL^−1^) from mycelial samples of a panel of fungal species comprising *L. biglobosa*, *Pyrenopeziza brassicae*, *Botrytis cinerea*, *Sclerotinia sclerotiorum*, *Alternaria brassicae*, *A. brassicicola*, *Verticillium longisporum* and *Zymoseptoria tritici* (=*Mycosphaerella graminicola*).

### PCR to screen for alleles of the AvrLm1 and AvrLm6 genes in mycelial samples

End-point PCR amplifications of avirulent allele *AvrLm1* and *AvrLm6* of *L. maculans* were carried out by the methods described above. The PCR primers (Van de Wouw et al. 2010) used were AvrLm1qF (5′-GGGTGTTTACTTCGCCTCAC-3′), AvrLm1qR (5′-ACGTTGTAATGAGCGGAACC-3′), AvrLm6qF (5′- TATTGGACAAAAGCCGAAGG-3′) and AvrLm6qR (5′-GCGAGAAGCAAGTGGAATGT-3′). PCR amplification was done in MJ Research PTC 200 Thermal Cycler (MJ Research Inc., Canada) in the total volume of 10 μL which contained 1 μL extracted DNA solution (10–100 ng uL^−1^), 0.2 mM dNTPs, 1 μM forward primer 1 μM 1 μM reverse primer, 0.5 U DNA DreamTaq polymerase (Thermo Fisher Scientific Inc. USA), 1 μL PCR buffer (10×, with Mg^2+^ 20 mM) and sterilized water was added to the total volume of 10 μL. PCR mixture was covered with 20 μL mineral oil. PCR conditions were as follows: 2 min at 94 ºC, followed by 45 cycles of 30 s at 94 ºC, 30 s at 58 ºC and 60 s at 72 ºC, with a final extension of 5 min at 72 ºC. PCR products were separated on a 2.0 % agarose gel, stained with ethidium bromide and visualized with UV light. To preclude negative detections being regarded as arising from PCR inhibition, duplex reactions were done in all cases along with the detection of minisatellite *MinLm2452*, which yielded two sizes of amplicons; a 103 bp product as the more prominent variant and a second less frequent 82 bp amplicon (Jedryczka et al. 2010a). The primers that were used for this amplification were MinLm2452F (5′-GTACATGGGCGGACAGGC-3′) and MinLm2452R (5′-CATTTACACTGCACACCTGCTCA-3′). The positive control was DNA from *L. maculans* isolate POL85 containing both *AvrLm1* and *AvrLm6* alleles. The negative control was water with no target DNA. A second negative control with target DNA template from *L. maculans* isolate PAW10 containing *avrLm1* and *avrLm6* alleles was included. This isolate lacked the expected avirulence allele PCR products but had minisatellite *MinLm2452*.

### Statistical analyses

DNA yields were correlated against the corresponding quantities of avirulence alleles of *AvrLm1* and *AvrLm6*, and also of *erg11*, with extracted spore tapes using Statistica version 9.0 (StatSoft Inc.). Due to non-linearity and non-normality of data for amounts of DNA of *L. maculans* a Spearman’s rank correlation was used to examine the relationships between the two measurement parameters.

## Results

**Fig. 1:**
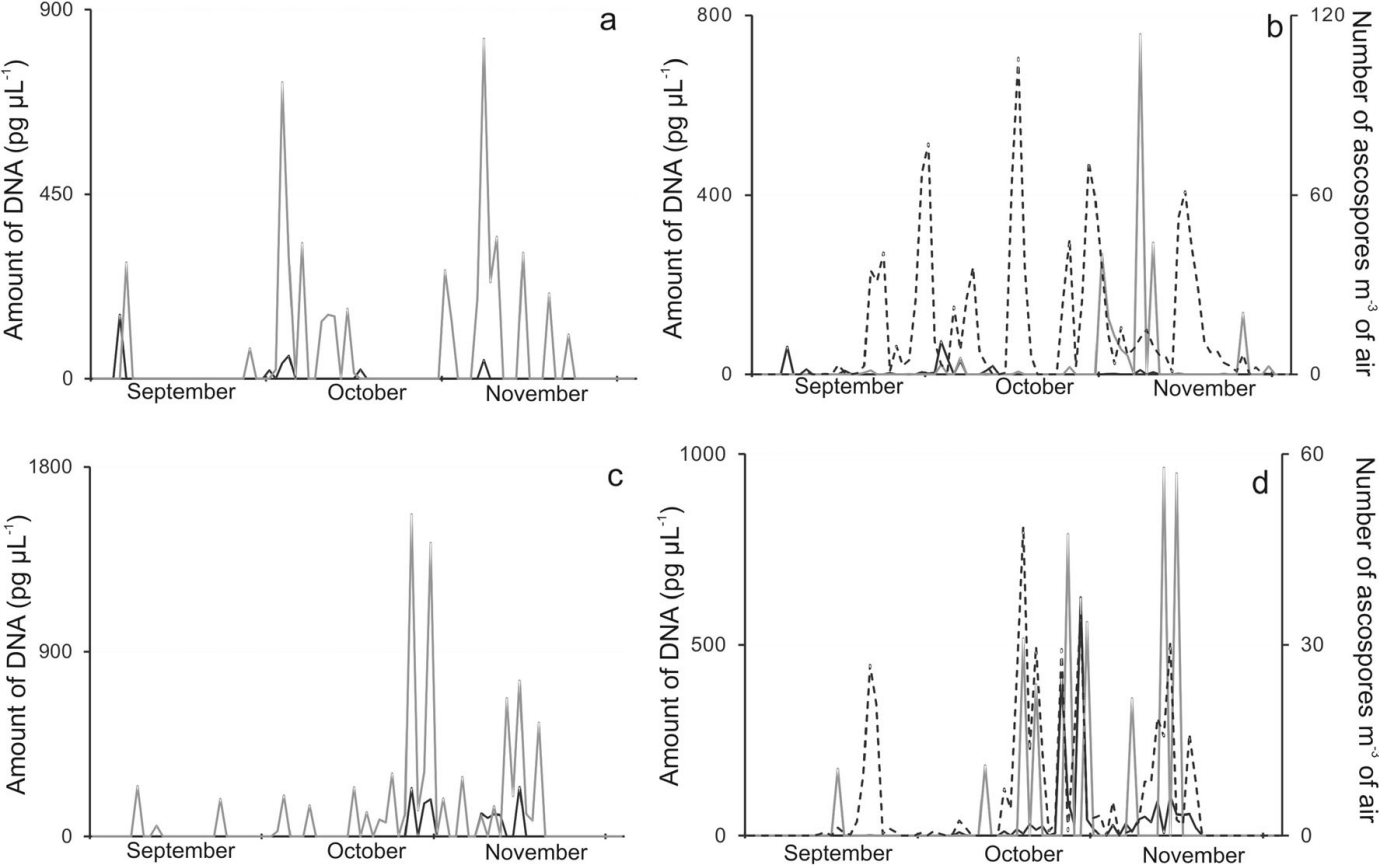
Seasonal fluctuations in DNA quantities or ascospore counts obtained by sampling airborne propagules collected over two autumns (**a**, **b**; 2006 and **c**, **d**; 2008) periods on Melinex tapes of a Burkard 7-day volumetric air sampler located in Poznan, Poland. Quantitative PCR assays detected avirulence alleles *AvrLm1* (**a**, **c**; *black line*), *AvrLm6* (**a**, **c**; *grey line*) or with primers based on *β*-tubulin (**b**, **d**; *black line*) or e*rg11* (**b**, **d**; *grey line*) fragments in the propagules of *Leptosphaeria maculans*. Ascospore release patterns (**b**, **d**; *dotted line*) were determined by light microscopic counts of *Leptosphaeria*-like ascospores per m^3^ of air

**Fig. 2:**
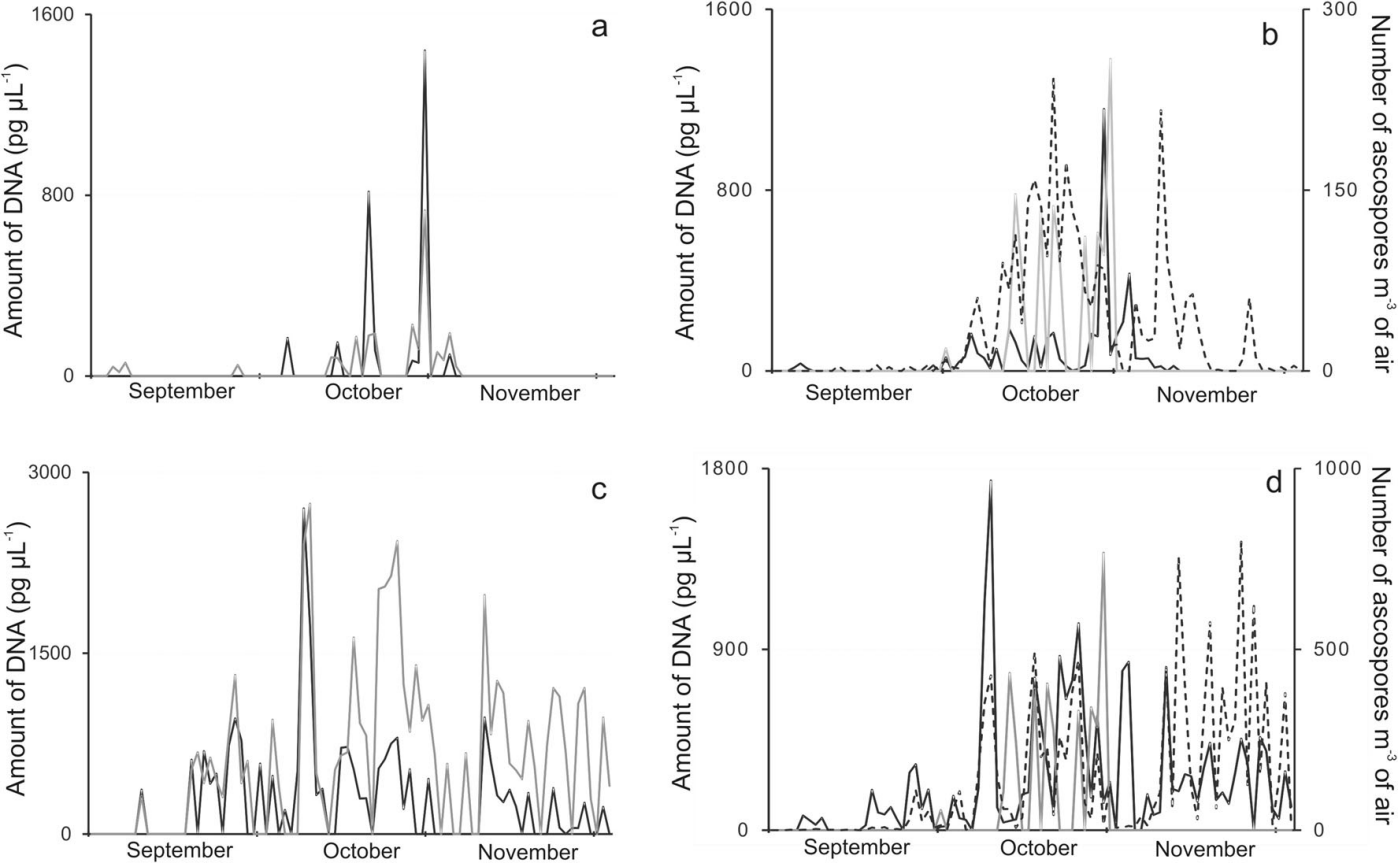
Seasonal fluctuations in DNA quantities or ascospore counts obtained by sampling airborne propagules collected over two autumns (**a**, **b**; 2002 and **c**, **d**; 2006) periods on Melinex tapes of a Burkard 7-day volumetric air sampler located in Rothamsted Research, Harpenden, UK. Quantitative PCR assays detected avirulence alleles *AvrLm1* (**a**, **c**; *black line*), *AvrLm6* (**a**, **c**; *grey line*) or with primers based on *β*-tubulin (**b**, **d**; *black line*) or e*rg11* (**b**, **d**; *grey line*) fragments in the propagules of *Leptosphaeria maculans*. Ascospore release patterns (**b**, **d**; *dotted line*) were determined by light microscopic counts of *Leptosphaeria*-like ascospores per m^3^ of air

Quantitative PCR analyses enabled the detection, separation and evaluation of avirulence alleles *AvrLm1* and *AvrLm6* in captured propagule samples thereby permitting a visualization of the patterns of the seasonal fluctuations of these fungal effectors across different years and experimental sites. In Poznan, Poland (Fig. 1) *AvrLm6* was predominant while *AvrLm1* was detected only at comparatively low levels in both 2006 and 2008. In the UK, in the autumn of 2002, however, *AvrLm1* allele predominated over *AvrLm6* in Harpenden (Fig. 2a). As shown in Table 1, in that year, the *AvrLm1* titre was at least 1.2 times greater than that for *AvrLm6*. Four years later, in 2006, the *AvrLm6* allele became over two times more prominent than *AvrLm1* in the qPCR assays of UK samples (Fig. 2c). The correlation coefficient between DNA concentrations of *AvrLm1* and *AvrLm6* was 0.712 (Table 1), and it ranged from 0.268 for data obtained in Poznan in 2006 to 0.859 for Rothamsted Research 2002. The relatively low correlation for data obtained in Poznan in 2006 appeared to arise from the very low *AvrLm1* allele titres, but corresponded nevertheless to the much higher frequency of *AvrLm6* DNA in these autumnal Polish air samples as compared to *AvrLm1*. In Poland *AvrLm6* allele was on average 8.5 times more frequent than *AvrLm1* in the 2 years studied, and the ratio ranged from 7:1 to 15:1 in favour of *AvrLm6*. It is noteworthy that the correlation between the qPCR-based detection of avirulence genes and the visual spore count was highly significant (*r* = 0.954; *P* > 0.001).

**Table 1:** Comparison of scores from light microscopic counts of *Leptosphaeria*-like ascospores, qPCR detection of DNA from *L.  maculans* spores and of avirulence alleles in air samples from Poland or the UK

Location x year	Spore count	DNA variant detected in air sample (pg)		Correlation *AvrLm6*:*AvrLm1*	Ratio *AvrLm6*:*AvrLm1*
		*AvrLm1* allele	*AvrLm6* allele		
Rothamsted 2002	2942	2911.2	2399.9	0.859	0.82
Rothamsted 2006	11327	22536.5	51040.0	0.684	2.26
Poznan 2006	1224	338.6	4989.6	0.268	14.74
Poznan 2008	421	1228.2	8306.0	0.724	6.76
Mean	3978.5	6753.6	16683.9	0.634	6.15

In both Poland (Figs. 1a–d) and the UK (Figs. 2a–d) fluctuations in the abundance of these *L. maculans*–specific avirulence alleles occurred in consonance with the seasonal patterns of ascospore release as judged by light microscopy and were also in agreement with patterns of fluctuations in the quantities of *L. maculans* DNA determined by qPCR assays. Judging by the titres of *erg11* from *L. maculans*, ergosterol biosynthetic activity was more easily measurable in airborne propagules from Poznan in 2008 than in 2006 (Figs. 1b and d). As shown in Fig. 2b and d, however, this activity was more easily quantified in captured air spora from the UK in both 2002 and 2006 and the fluctuation patterns had maximal prominence in the October of both years.

**Fig. 3:**
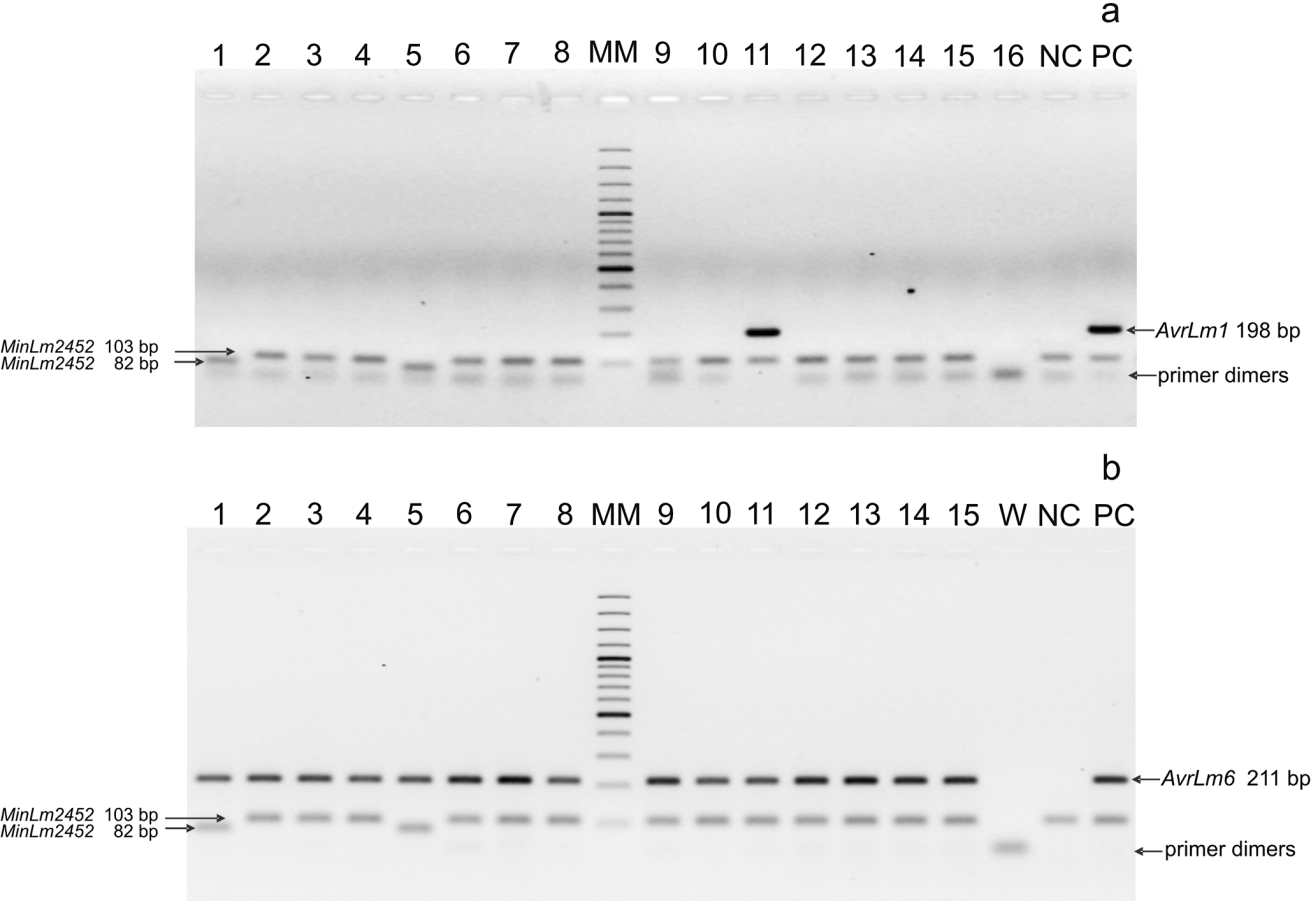
Detection of avirulence alleles *AvrLm1* (**a**) and *AvrLm6* (**b**) in isolates (lanes 1 to 15) of *Leptosphaeria maculans* obtained from infected plants of oilseed rape grown in Pawlowice, central-west Poland. W – water, NC – negative control (DNA of the isolate PAW10 containing *avrLm1* and *avrLm6*), PC – positive control (DNA of the isolate POL85 containing *AvrLm1* and *AvrLm6*)

For avirulence alleles, a similar data set (Table 2) was obtained *in planta*, from bioassays done on infected cotyledonary leaves of *Brassica napus* employing molecular detection of either *AvrLm1* or *AvrLm6* by end-point PCR with allele-specific primers (Fig. 3). Out of the 51 isolates of *L. maculans* that were sampled randomly from fields in Great Poland (within which the Burkard volumetric spore trap was located) only one isolate, obtained from an oilseed rape field in Pawlowice (Fig. 3a, sample 11), was avirulent on the oilseed rape differential cultivar (cv. Columbus) with the corresponding *Rlm1* resistance gene. All the remainder were virulent on this cultivar, i.e. possessed the *avrlm1* allele. In contrast, all tested isolates possessed *AvrLm6* avirulence allele (Fig. 3b).

**Table 2:** Frequency (%) of avirulence alleles in populations of *Leptosphaeria maculans* isolates from infected cotyledonary leaves of oilseed rape from Great Poland region in central-west Poland

No.	Location	Cotyledon test				Molecular detection	
		No. of isolates	*AvrLm1* allele	*AvrLm6* allele	*AvrLm9* allele	*AvrLm1* allele	*AvrLm6* allele
1	Cerekwica	11	0	100	0	0	100
2	Gora	4	0	100	0	0	100
3	Nowa Wies Ujska	20	0	100	0	0	100
4	Pawlowice	16	6.25	100	0	6.25	100
Mean		51	1.96	100	0	1.96	100

## Discussion

The results of this study demonstrate a successful derivation of biological information, beyond earlier description and evaluation of numerical abundance, from season-long screening and monitoring of air borne fungal pathogen spores. Quantitative PCR techniques were employed for the detection of the *L. maculans* avirulence alleles *AvrLm1 and AvrLm6* in DNA extracted from airborne particles on tapes from spore traps that were operated in Poland and the UK within oilseed rape fields over the autumn of 2002, 2006 and 2008. The patterns of *Leptosphaeria*-like ascospore release were also assessed on stained Melinex tapes by light microscopy and fluctuations in pre-harvest aerial biomass of *L. maculans* inoculum were determined by real-time qPCR. In this study, the primers that were designed for *AvrLm1*and *AvrLm6* detection recognized, respectively, a 301 bp (nucleotides 109–409) and a 307 bp (nucleotides 353–659) fragment of the open reading frames of these avirulence effectors. Primers recognizing a 198 bp (nucleotides 212–409, for *AvrLm1*) and a 211 bp (nucleotides 328–479, for *AvrLm6*) gene fragment were used instead by Van de Wouw et al. (2010) to determine and compare by qPCR the proportions of *AvrLm1* and *AvrLm6* alleles in airborne ascospores of *L. maculans* that were captured from Rothamsted Research with their ratio on infected plants sampled from farmer’s fields of oilseed rape in the same area. The latter primers found application in the current study in the end-point PCR screening of infected oilseed rape plants for *AvrLm1* and *AvrLm6* alleles.

The correlations between the ascospore counts and concentrations of *L. maculans* DNA in captured air spora in this study were highly significant, suggesting the possibility of replacing the laborious and time consuming visual assessments done by staining and light microscopic counts with molecular biological detection. The latter approach furthermore provides opportunities for generating additional informative data. Evidence for ergosterol biosynthetic activity (as judged by fluctuations in the titre of fragments of *erg11* from *L. maculans*) was conceived in the current study as a test for spore viability potential that could also assist in future detection of and screens for mutations affecting sensitivity to azole fungicides. Evidence is currently lacking in *L. maculans* (Huang et al. 2011) for mutations in this *Cyp51* gene encoding the azole target sterol 14α-demethylase.

Avirulence alleles of *L. maculans* code for small secreted protein effectors which play a putative role in modulating innate immunity in oilseed rape, the major brassica host plants (Rouxel and Balesdent 2010). Genes coding for *AvrLm1*, *AvrLm4*-*7* and *AvrLm6* have been cloned (Gout et al. 2006; Fudal et al. 2007). When constitutively or inducibly expressed at the onset of infection, recognition in the host plant by *Rlm1* and *Rlm6* of these products of *AvrLm1* and *AvrLm6* results in phoma disease resistance. Continual and widespread cultivation of oilseed rape cultivars with monogenic *Rlm1*-mediated resistance precipitated in France and Australia the rapid evolution of *avrLm1*-based virulence (through deletion mutation events within the *AvrLm1* locus; Gout et  al. 2007) in *L. maculans* populations, epiphytotic phoma stem canker disease spread and massive oilseed rape yield losses. Consequently, oilseed rape breeders and farmers in continental Europe and Australia switched to the cultivation of oilseed rape cultivars with multigenic and more durable resistance. In the current study, the avirulence gene *AvrLm1* was rarely detected in Polish air samples and only sporadically in plants, apparently subsequent to the ineffectiveness and impractical usefulness of *Rlm1* as resistance gene source. In contrast, *AvrLm6* allele was detected in most airborne *L. maculans* propagules and phoma-infected field-sown oilseed rape plants from Poland implying the sustained effectiveness of *Rlm6*-based stem canker resistance. This was evinced by a minimum of 8:1 average ratio in the frequencies of *AvrLm6*:*AvrLm1* alleles across the autumn of 2006 and 2008. In the UK, however, the frequency ratios of *AvrLm6*:*AvrLm1* alleles was less polarized, and even though *AvrLm6* was more frequently detected, the proportion between these avirulence alleles was about 2:1. It may be concluded that in the UK, the *AvrLm1* allele is present in air samples approximately four times more often than in Poland. Annually, a varied number of genetically heterogeneous oilseed rape cultivars and lines are sown to field experimental plots at Rothamsted Research; it is reasonable that DNA from *L. maculans* propagules captured by traps operated at this UK site around the debris of these cultivars would reflect the greater diversity obtained in the current study. In general, data obtained in Poland established that *AvrLm6* allele was present in all or most DNA samples obtained from ascospores of *L. maculans*, whereas *AvrLm1* was nearly non existent. It is clear from our study that no spores with *avrLm6* in the air resulted from no plants infected with isolates of *L. maculans* possessing *avrLm6* allele, what in turn resulted in no inoculum with *avrlm6*.

Knowledge on variation of avirulence genes in *L. maculans* populations in Poland dates back to 2000–2001, when a population of 92 isolates originating from different parts of the country was studied using a cotyledon test, followed by PCR with *Vir1* marker (Jedryczka 2007). The population in those years was found to be monomorphic with respect to *AvrLm7* (avirulent genotype) and *avrLm2*, *avrLm3* and *avrLm9* (virulent genotypes) but polymorphic for *AvrLm1* (58.7 %) and *AvrLm4* (3.3 %). The interaction phenotype agreed with molecular study (90.2 %). The most recent Europe-wide monitoring, concerning the occurrence of avirulence genes was done by Stachowiak et al. (2006) at six experimental sites across the main oilseed rape growing regions embracing Poland (two sites), the UK (two), Germany (one) and Sweden (one). In total, 603 isolates were collected during the autumn of 2002 (Germany and the UK) and 2003 (Poland and Sweden). No isolates had the virulence allele *avrLm6* and three virulence alleles (*avrLm2*, *avrLm3* and *avrLm9*) were present in all isolates. The isolates were polymorphic for *AvrLm1*, *AvrLm4*, *AvrLm5* and *AvrLm7* alleles, with virulence alleles at *AvrLm1* and *AvrLm4* loci and avirulence alleles at *AvrLm7* and *AvrLm5* loci predominant in populations. Virulent *avrLm7* isolates were found at only one site in Sweden. Approximately 90 % of all isolates belonged to one of two races, Av5–6–7 (77 % of isolates) or Av6–7 (12 %). The results were comparable to those of a similar survey done in France in autumn 2000 and 2001 (Balesdent et al. 2005) which reported that resistant genes *Rlm6* and *Rlm7* were still effective against *L. maculans* in oilseed rape in Europe.

The current study on avirulence alleles showed that the situation in the Poznan area of Poland had not deviated in the 3–5 years from a previous monitoring work that was done in 2003, and that *AvrLm6* is still present in isolates of *L. maculans*, whereas *AvrLm1* is nearly absent in the pathogen’s population. Moreover the situation in the UK with *AvrLm1* avirulence gene was similar to that reported by Stachowiak et al. (2006), with nearly 20 % of the isolates avirulent on cultivars possessing *Rlm1* resistance gene. In the current work we have also established that *AvrLm1* isolates are on average four times more frequent in the UK, in comparison to Poland. This screen suggested, however, a higher proportion of isolates virulent to oilseed rape lines with *Rlm6* resistance gene than found in Rothamsted in 2001 by Stachowiak et al. (2006). A large survey of *L. maculans* isolates from Australia, Canada and South America has also shown that most isolates possessed the *AvrLm6* avirulence allele (Dilmaghani et al. 2009). The annual, seasonal and regional variations might originate in selective pressures dictated by cultivar choice as well as other unforeseen reasons, such as the composition of brassicaeous weeds, with different resistance genes from those found in crucifers. The study suggests that *Rlm6* resistance gene could provide effective resistance to *L. maculans* in Poland. However, in the UK, the resistance appears to be under threat. The deployment of *Rlm6* resistance cultivars of oilseed rape should when effected be carefully managed by supplementation with qualitative resistance that will prevent rapid stem canker resistance erosion that was witnessed with *Rlm1* in France (Rouxel et al. 2003) and in Australian *B. napus* cultivar Surpass 400 (Li et al. 2003) with ‘*sylvestris*-derived’ resistance.

The results obtained in the current study confirm quantitative real-time PCR as an accurate and reliable technique due to its enormous sensitivity, possibility of detection of reaction progress, speed of analyses and precision permitting the examination of pico- and even femto-gram differences between spore and effector samples. Additional use of reference genes, such as *actin* in plant stress-related studies makes it even more reliable (Kozera and Rapacz 2013). The current study further demonstrates that methods employed for screening avirulence genes in air samples (Van de Wouw et al. 2010) could be successfully employed for routine larger-scale and epidemiological studies of plant pathogen populations (Heard and West 2014; West 2014), yielding deeper insights into the avirulence allele compositions and race structures of wind- and rain-dispersed pathogens in particular. At the limits of the powers of the application of qPCR, opportunities exist with other approaches (such as high throughput pyrosequencing; Van de Wouw and Howlett 2012) for molecular biological analyses of other avirulence and effector genes in *L. maculans* propagules captured on spore tapes. Integrated plant protection guidelines, obligatory for all European Union Member States since early 2014 (Directive 2009/128/EC), stipulate the restriction of chemical sprays of agricultural crops to real needs in specific seasons and locations, based on monitoring results and threshold levels. Taken together, molecular biological detection of gene variants, when combined with various early detection techniques such as those based of aerobiology, are a powerful tool that can be employed for low-, as well as, non-chemical, early and regular interventions in integrated pest management.
